# Genetic algorithm supported by graphical processing unit improves the exploration of effective connectivity in functional brain imaging

**DOI:** 10.3389/fncom.2015.00050

**Published:** 2015-05-05

**Authors:** Lawrence Wing Chi Chan, Bin Pang, Chi-Ren Shyu, Tao Chan, Pek-Lan Khong

**Affiliations:** ^1^Department of Health Technology and Informatics, Hong Kong Polytechnic UniversityHong Kong, China; ^2^Informatics Institute, University of MissouriColumbia, MO, USA; ^3^Department of Diagnostic Radiology, University of Hong KongHong Kong, China

**Keywords:** genetic algorithms, magnetic resonance imaging, structural equation modeling, graphical processing unit, effective connectivity, path model, neuronal circuitry

## Abstract

Brain regions of human subjects exhibit certain levels of associated activation upon specific environmental stimuli. Functional Magnetic Resonance Imaging (fMRI) detects regional signals, based on which we could infer the direct or indirect neuronal connectivity between the regions. Structural Equation Modeling (SEM) is an appropriate mathematical approach for analyzing the effective connectivity using fMRI data. A maximum likelihood (ML) discrepancy function is minimized against some constrained coefficients of a path model. The minimization is an iterative process. The computing time is very long as the number of iterations increases geometrically with the number of path coefficients. Using regular Quad-Core Central Processing Unit (CPU) platform, duration up to 3 months is required for the iterations from 0 to 30 path coefficients. This study demonstrates the application of Graphical Processing Unit (GPU) with the parallel Genetic Algorithm (GA) that replaces the Powell minimization in the standard program code of the analysis software package. It was found in the same example that GA under GPU reduced the duration to 20 h and provided more accurate solution when compared with standard program code under CPU.

## Introduction

Human brain regions are activated in response to external stimuli or in carrying out cognitive tasks. Functional MRI is an imaging modality that can interrogates the hemoglobin oxygenation and deoxygenation related to blood flow at the activated regions through the change in magnetic signals. The activated brain regions can be identified by their synchrony with the applied stimuli or the tasks that are being carried out by the human subject (Erkonen and Smith, 2009). The functional imaging technique becomes more crucial for understanding the regional brain functions and the neuronal connectivity in response to the external factors. Brain regions are represented by nodes connected with links in a brain network. Depending on the scope of study, the links could represent anatomical, functional or effective connectivity. Anatomical links are usually used for studying white matter tracts among brain regions. Functional links implicate that the brain regions exhibit strong temporal association between the detected regional signals. Effective links are directional and indicate if a region influences another directly or indirectly. Based on the existing knowledge, anatomical links hypothesize a brain network model concerning a relatively small number of regions. Using the time series of brain dynamics acquired by functional imaging technique, such as fMRI, effective links can be inferred from the brain network model by quantifying the directional connectivity strength (Rubinova and Spornsd, 2010).

### Structural equation modeling

Structural equation modeling (SEM) is a mathematical tool for computing the effective connectivity based on the functional brain imaging data. SEM is aimed to infer a path model for quantifying the strength of the interregional directed connections with the associated path coefficients. The path model is formulated by the anatomical constraints extracted from the existing studies and knowledge and the values of path coefficients are obtained by data-driven path analysis. Prior to the path analysis, we need to specify the regions of interest (ROI's) that are potentially activated due to the stimuli. The data pre-processing steps include denoising, trend removing, motion correction, normalization, and deconvolution using hemodynamic response function (Stein et al., 2007).

In SEM, an interregional correlation matrix is derived using the processed time series of the ROI's. The estimated correlation matrix and the adjustable path coefficients constitute the maximum likelihood (ML) discrepancy function. With a fixed estimate of correlation matrix, the “best” path model is characterized by path coefficients that minimize the ML discrepancy function. The minimization process is performed iteratively where the number of unconstrained path coefficients increases after each constrained minimization of ML discrepancy function (Bullmore et al., 2000). As the time required for such computation increases geometrically with the number of the path coefficients, only a few brain regions were considered in the previous studies. Quad-Core Central Processing Unit (CPU) takes a whole day to compute a single iteration for estimating 40 path coefficients. Duration of a month is required for iterations from 0 to 40 path coefficients.

### Supercomputing solution

Graphics Processing Unit (GPU), equipped with dedicated pixel processing hardware, could speed up the arithmetic computation. Software development tools, e.g., Common Unified Device Architecture (CUDA) toolkit (http://www.nvidia.com/), transform the scientific computing platform from CPU to GPU (Ritchie and Venkatraman, 2010). The current applications of GPU are mostly DNA and protein sequence alignments in bioinformatics. Eklund et al. (2012) and Eklund et al. (2014) used GPU to analyse the associations between brain regions but the directed connections cannot be identified using such functional analysis. GPU has been applied for analyzing the effective connectivity in human brain (Chan et al., 2013). However, the performance of GPU in the minimization process has not been compared with that of CPU for fixed periods of computational time and the accuracy of the identified connections has not been validated against the published findings.

Genetic Algorithm (GA) is a parallel algorithm for randomly exploring the “best” solution with a cost function. A number of successful applications for porting GA to GPU have been demonstrated previously (Wang and Shen, 2012). As an alternative to the parallel iterations of GA, Simulated Annealing (SA) controls the iteration convergence through a cooling schedule. GA and SA are integrated to form the hierarchical parallel genetic simulated annealing (HP-GSA) algorithm under GPU (Mahfoud and Goldberg, 1995). This study demonstrates the powerful application of HP-GSA under GPU in accurately analyzing fMRI data with much lower computational load.

## Methods

### Subjects and data acquisition

An fMRI study recruited 11 subjects who were university students, 21–32 years old and early Chinese-English bilinguals. The image data were acquired from the subjects using a 1.5T scanner with T2^*^-weighted gradient-echo EPI sequence. English verbs, English nouns, Chinese verbs and Chinese nouns were presented to the subjects through an LCD projector system during the fMRI scan (Chan et al., 2008).

The dataset is comprised of dynamic three-dimensional (3D) voxel values collected by the fMRI scan. The regional signals were obtained by averaging the time series of voxels over the regions. We denote the number of subjects and the number of data points of time series by *m* and *n*, respectively where *m* is generally much less than *n*. Let *R_i_* be an *m* × *n* matrix containing the time series data for a given *i*^th^ region, whose values have already been standardized to zero mean and unit variance.

### Mathematical principle of neuronal connectivity analysis

In each region across all subjects, the dominant component of time series due to the stimuli was identified by Principal Component Analysis (PCA). The singular value decomposition yields the data matrix given by,

(1) $$R_{i}=U_{i}L_{i}V_{i}^{T}$$

where the columns of *V_i_* represent the eigentimeseries (eigenvectors) of *R_i_* and the diagonal elements of *L_i_*, the square roots of the eigenvalues of *R_i_*, denoted by λ_1_, λ_2_, …,λ_m_.

The first principal component of *R_i_*, denoted by *v_i_*(*t*), drives the statistical variation of regional time series subject to the stimuli-induced neuronal interactions. We denote the number of ROI's by *p* and the first principal components of *p* ROI's at time *t* by *v*(*t*), a *p*×1 vector [*v*_1_(*t*), *v*_2_(*t*), …, *v_p_*(*t*)]^T^. The path model can be represented by a set of simultaneous regression equations given by,

(2) v=Kv+u

where *K* represents a *p* × *p* matrix of path coefficients with zero diagonal elements and *u*, the vector of residual time series independent of the neuronal interactions. If there is no evidence of direct anatomical connectivity of a path, its off-diagonal elements of *K* will be set to zeros. We define the interregional correlation matrix as *C*=E[*vv*^T^]. With the estimated path model the parametric estimate of *C*, given by *C*_1_, can be obtained by the following formula,

(3) $$C_{1}={\left(1-\hat{K}\right)}^{-1}R{\left(1-\hat{K}\right)}^{-T}$$

where *R*=E[*uu*^T^] represents a *p* × *p* diagonal matrix of the residual variances, *r*_1_, *r*_2_, …, *r_p_*, which are given by,

(4) $$r_{i}=1-\frac{\lambda_{1}^{2}}{\sum_{j=1}^{m}\lambda_{j}^{2}}$$

If the *q* × 1 vector containing the non-zero path coefficients in *K* is denoted by θ, the matrix *C*_1_ will become a parametric function of path coefficients θ. Another estimate of *C*, denoted by *C*_2_, can be calculated by the observed correlation matrix of the first principal components, E[*vv*^T^]. According to SEM, the values of θ governing the path model are estimated by minimizing the maximum likelihood (ML) discrepancy function *F* given by,

(5) $$F\left(C_{1}\left(\theta \right),C_{2}\right)=\log\left|C_{1}\left(\theta \right)\right|+tr\left(C_{2}C_{1}^{-1}\left(\theta \right)\right)-\log\left|C_{2}\right|-p$$

### Iterative minimization under CPU and GPU

An automated search method was applied as the number of paths or path coefficients, *q*, for the best fitting model is unknown. The automated search process starts with a null model where all non-zero path coefficients θ are constrained to zeros. Lagrangian multiplier (LM) associated with each constrained coefficient in θ is then computed. In the next iteration, the coefficient with the largest LM becomes unconstrained in minimizing *F*. LM is then computed for the second unconstrained coefficient and *F* is minimized again against the two unconstrained coefficients. The iterations continue until the parsimonious fit index of the path model reaches a pre-specified acceptable value (Bullmore et al., 2000).

The minimization of F is very fast in the first few iterations due to the small number of the unconstrained path coefficients. However, the computing load increases in geometric order with the number of unconstrained path coefficients. The number of possible paths will be 30 if six ROI's are considered. As we limited the number of non-zero path coefficients to twelve, there are _30_C_12_ = 86,493,225 possible solutions. If we use the AMD Phenom X4 9850 Quad-Core CPU at 2.5 GHz and 2MB cache memory, the optimization of each possible solution takes 0.1 s and the best solution is obtained in 3 months. To make the path model estimation a feasible approach, we rewrote the coding for minimization process under CPU into that under NVIDIA Tesla C2050 GPU card equipped with 448 cores at 1.15 GHz and 3 GB global memory. Each GPU card can theoretically operate with up to 30,000 threads in parallel. A computer equipped with four GPU cards can operate with 120,000 threads. With the GPU approach, it will take 2 h only to get the best from 86,493,225 possible solutions.

Analysis of Functional NeuroImages (AFNI), a C program set for analyzing and visualizing of fMRI data of human brain, was used in this study (Cox, 1996). In the original AFNI source code, the iterative minimization of F is implemented by the modules “1dSEM” and “powell_int” where Powell Optimization approach is applied. Since GPU could not accelerate the Powell Optimization routine, we replaced the routine by HP-GSA for GPU implementation (Mahfoud and Goldberg, 1995).

## Results

We analyzed the effective connectivity among six ROI's, including left middle frontal gyrus BA9 and BA10 (LMFG1, LMFG2), left medial frontal gyrus BA6/8 (LMedFG), left inferior parietal lobule BA39/40 (LIPL), left cerebellum (LCere) and left inferior frontal gyrus BA45/46 (LIFG) for comparing the performance of 1dSEM and HP-GSA. There were altogether 30 possible paths. The number of non-zero path coefficients was limited to 12.

The computation of standard “1dSEM” was performed under Quad-Core CPU, while that of the modified HP-GSA module was done under GPU. Since it has been proved that the computing speed of GPU is about 1000 time faster than that of CPU, we were particularly interested in their performance in minimizing F values and identifying accurate neuronal connections.

With a setting of the population size 1024, for individuals composed of 6 ROI's and a maximum generation number 200, HP-GSA obtained the best θ that minimizes the function F. A fixed reference F value was obtained by the standard “1dSEM.” We performed 100 trials and grade the performance of HP-GSA according to the following criteria.

[Improved:]        F_HP−GSA_ < F_1dSEM_
[Worsened:]        F_HP−GSA_ ≥ F_1dSEM_

**Figure 1:**
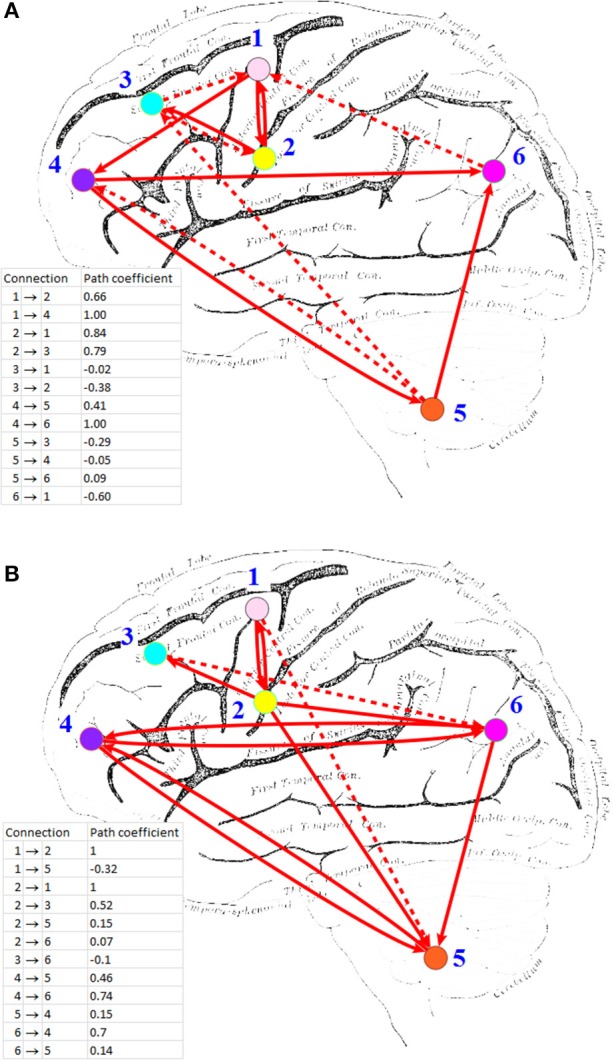
**Effective connectivity maps inferred by (A) 1dSEM under CPU, and (B) HP-GSA under GPU**. ROI's: (1) L medial frontal gyrus; (2) L inferior frontal gyrus; (3) L middle frontal gyrus (BA 9); (4) L middle frontal gyrus (BA 10); (5) L cerebellum; (6) L inferior parietal lobule. Solid line indicates positive path coefficient and dotted line indicates negative path coefficient. Background picture was obtained from http://imgkid.com/left-brain-diagram.shtml.

HP-GSA yielded better minimization results in 56 trials and worse results in 44 trials. Although the improved performance of HP-GSA was seemingly unremarkable, we could perform the HP-GSA minimization process repeatedly and choose the lowest F value. If we repeat the HP-GSA minimization process for 10 times, the probability for getting worse results is 0.44^10^, i.e., 0.027%. HP-GSA under GPU took only 20 h to achieve 99.973% confidence in getting a minimization solution better than the 1dSEM under CPU could achieve in 3 months. The neuronal connections of the best solutions obtained from 1dSEM and HP-GSA with 100 trials are illustrated in Figures 1A,B respectively and are compared in Table 1 with the available literature support.

**Table 1:** **Comparison between 1dSEM under CPU and HP-GSA under GPU in exploring effective connectivity**.

**No**.	**ROI1**	**ROI2**	**1dSEM under CPU**	**HP-GSA under GPU**	**References**
1	L medial frontal gyrus	L cerebellum	No connection	ROI 1→ ROI 2	Sundermann and Pfleiderer, 2012
2	L inferior frontal gyrus	L cerebellum	No connection	ROI 1→ ROI 2	Booth et al., 2007
3	L inferior frontal gyrus	L inferior parietal lobule	No connection	ROI 1→ ROI 2	Menenti et al., 2012
4	L middle frontal gyrus (BA 9)	L inferior parietal lobule	No connection	ROI 1→ ROI 2	Not available
5	L inferior frontal gyrus	L middle frontal gyrus (BA 9)	ROI 1↔ ROI 2	ROI 1→ ROI 2	Ardila et al., 2015
6	L middle frontal gyrus (BA 10)	L inferior parietal lobule	ROI 1→ ROI 2	ROI 1↔ ROI 2	Not available
7	L middle frontal gyrus (BA 10)	L cerebellum	ROI 1↔ ROI 2	ROI 1↔ ROI 2	Heisterueber et al., 2014
8	L medial frontal gyrus	L inferior frontal gyrus	ROI 1↔ ROI 2	ROI 1↔ ROI 2	Not available
9	L inferior parietal lobule	L cerebellum	ROI 1← ROI 2	ROI 1→ ROI 2	Not available
10	L medial frontal gyrus	L middle frontal gyrus (BA 10)	ROI 1→ ROI 2	No connection	Not available
11	L middle frontal gyrus (BA 9)	L medial frontal gyrus	ROI 1→ ROI 2	No connection	Not available
12	L cerebellum	L middle frontal gyrus (BA 9)	ROI 1→ ROI 2	No connection	Not available

## Discussions and conclusion

To apply GPU for computing the effective connectivity, the standard SEM algorithm was replaced by the GA optimization routine. It was clearly shown that the optimization process of GA under GPU was much faster than that of the standard SEM under CPU. This study also compared the performance of HP-GSA under GPU against that of 1dSEM under CPU in iteratively minimizing the ML discrepancy function and accurately identifying the neuronal connections. It was found in 56% of trials that ML discrepancy function minimized by GA was lower than that by standard SEM algorithm. Although the difference between the values of ML discrepancy function minimized by the two approaches was not significantly large, the individual connections between the ROI's derived from the solutions exhibited largely different effective connectivity maps. It is critical to evaluate the accuracy of the identified neuronal connections against the ground truth supported by the published findings.

The best solutions obtained from 1dSEM and HP-GSA yielded two different connectivity maps that are illustrated in Figures 1A,B, respectively and compared in Table 1. A rigorous literature search through PubMed and BrainMap was performed to validate the obtained connections with the available published findings (Fox et al., 2005). HP-GSA identified connections 1-4 but 1dSEM did not. Cognitive control, including task switching, involves the inferior frontal junction (IFJ) area that was found to be significantly co-activated with medial frontal gyrus and cerebellum in language processing (Sundermann and Pfleiderer, 2012). Since the English verbs, English nouns, Chinese verbs and Chinese nouns were presented to the subjects, the processes included the switching between English and Chinese and that between verb and noun. The connection 1 is therefore justified by this published finding. In a functional magnetic resonance imaging study, the neural connectivity in adults was analyzed using dynamic causal modeling (Booth et al., 2007). It was shown that the cerebellum was connected to the left inferior frontal gyrus during the rhyming judgment, a language processing task. This finding justified the connection 2. A speaking experiment explored the sense of each subject upon the presentation of target pictures preceded by its corresponding verb and the request for describing the target pictures (Menenti et al., 2012). The sense was reflected by the neural connectivity map derived from functional magnetic resonance imaging. It was shown that the response in BA39 including inferior parietal lobule and in the left middle frontal gyrus increased after repetition of sense. The connection 3 is supported by this experimental result.

A behavioral study performed meta-analyses for assessing the language network, the visual perception network and their contrasts and convergence involving BA37 (Ardila et al., 2015). Significant connection between BA37, middle frontal gyrus (BA 9), and inferior frontal gyrus (BA45) was found among 12 identified clusters. The meta-analysis results supported the connection 5. The neural correlates underlying the individuals' variability in German word stress processing were investigated in a functional magnetic resonance imaging study. The neuroimaging evidence showed clusters of voxels co-activated in cerebellum and middle frontal gyrus (BA 10) bilaterally. These relevant published findings supported mostly the connections identified by the HP-GSA but not the 1dSEM. Thus, the HP-GSA under GPU is a highly recommended approach that outperformed 1dSEM under CPU in accurately identifying the biological truth of neuronal connectivity.

### Conflict of interest statement

The authors declare that the research was conducted in the absence of any commercial or financial relationships that could be construed as a potential conflict of interest.
